# Tri-*p*-tolyl­phosphine

**DOI:** 10.1107/S160053680802374X

**Published:** 2008-08-20

**Authors:** Hao Wang, Yi-Bin Wang, Bo-Nian Liu, Shi-Gui Tang, Ping Wei

**Affiliations:** a College of Life Sciences And Pharmaceutical Engineering, Nanjing University of Technology, Nanjing 210009, People’s Republic of China; b College of Science, Nanjing University of Technolgy, Xinmofan Road No.5, Nanjing 210009, People’s Republic of China

## Abstract

In the title compound C_21_H_21_P, the P atom is situated on a crystallographic threefold rotatory-inversion axis, resulting in threefold rotation symmetry of the title compound. The dihedral angles between the symmetry-related benzene rings are 87.40 (18)°.

## Related literature

For related literature, see: Brown *et al.* (1988 ▶).
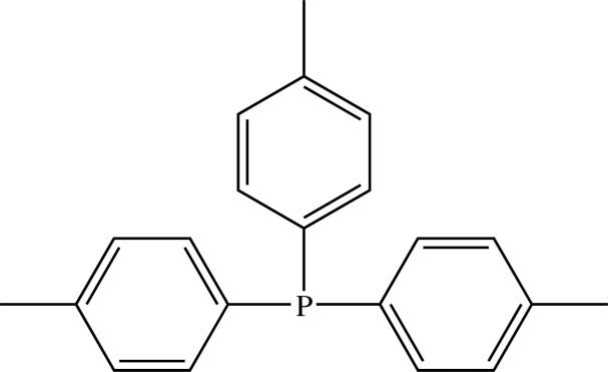


## Experimental

### 

#### Crystal data

C_21_H_21_P
*M* *_r_* = 304.35
Trigonal, 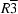

*a* = 12.6562 (18) Å
*c* = 19.696 (4) Å
*V* = 2732.2 (8) Å^3^
*Z* = 6
Mo *K*α radiation
μ = 0.15 mm^−1^
*T* = 293 (2) K
0.40 × 0.30 × 0.20 mm

#### Data collection

Enraf–Nonius CAD-4 diffractometer
Absorption correction: ψ scan (North *et al.*, 1968 ▶) *T*_min_ = 0.958, *T*_max_ = 0.971
3464 measured reflections
1095 independent reflections
790 reflections with *I* > 2σ(*I*)
*R*_int_ = 0.050
3 standard reflections every 200 reflections intensity decay: none

#### Refinement

*R*[*F* ^2^ > 2σ(*F* ^2^)] = 0.063
*wR*(*F* ^2^) = 0.171
*S* = 1.03
1095 reflections
67 parameters
H-atom parameters constrained
Δρ_max_ = 0.26 e Å^−3^
Δρ_min_ = −0.34 e Å^−3^

### 

Data collection: *CAD-4 Software* (Enraf–Nonius, 1989 ▶); cell refinement: *CAD-4 Software*; data reduction: *XCAD4* (Harms & Wocadlo, 1995 ▶); program(s) used to solve structure: *SHELXS97* (Sheldrick, 2008 ▶); program(s) used to refine structure: *SHELXL97* (Sheldrick, 2008 ▶); molecular graphics: *SHELXTL* (Sheldrick, 2008 ▶); software used to prepare material for publication: *SHELXL97*.

## Supplementary Material

Crystal structure: contains datablocks global, I. DOI: 10.1107/S160053680802374X/kj2091sup1.cif

Structure factors: contains datablocks I. DOI: 10.1107/S160053680802374X/kj2091Isup2.hkl

Additional supplementary materials:  crystallographic information; 3D view; checkCIF report

## Figures and Tables

**Fig. 1.**
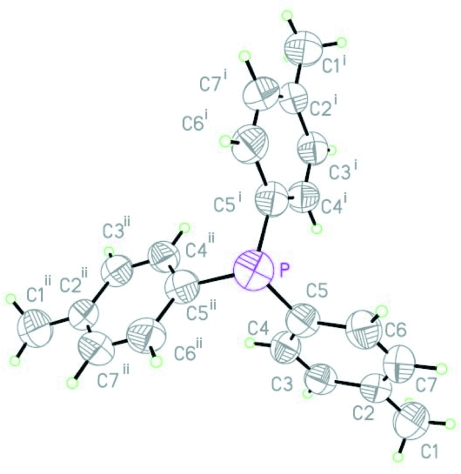
The molecular structure of the title compound, showing the atom-numbering scheme and displacement ellipsoids at the 30% probability level. Symmetry codes: (i) 1 - x + y,1 - x,z (ii) 1 - y + 1,x-y,z

## supplementary crystallographic information

### Comment

Some organophosphorus derivatives are important chemical materials, which are primarily used as intermediates of organic phosphorus flame retardants and phosphorus ligands in biphasic water soluble catalysts. The P atom is situated on a crystallographic threefold rotatory-inversion axis, resulting in threefold rotation symmetry of the title compound.

The dihedral angles between the symmetry-related benzene rings are 87.40 (18)°.

### Experimental

20 g Sodium (0.870 mol) was added to 125 ml toluene, then the mixture was heated up to 383 K and stirred to form fine particles of sodium, which subsequently melted. Then the temperature was lowered to 323 K. *P*-chlorotoluene (55.2 g / 0.436 mol) and phosphorus trichloride (19.8 g / 0.144 mol) were added, keeping the temperature between 323 K and 333 K for two hours. The product was concentrated in a vacuum to gain a white solid (35.0 g, 80%) (Brown *et al.*, 1988). The pure title compound was obtained by crystallizing from methanol. Crystals suitable for X-ray diffraction were obtained by slow evaporation of an methanol solution.

### Refinement

All H atoms bonded to the C atoms were placed geometrically at the distances of 0.93–0.97 Å, and included in the refinement in riding motion approximation with *U*_iso_(H) = 1.2 or 1.5 *U*_eq_ of the carrier atom.

### Crystal data

**Table:**

C_21_H_21_P	*Z* = 6
*M**_r_* = 304.35	*F*_000_ = 972
Trigonal, *R*3	*D*_x_ = 1.110 Mg m^−^^3^
Hall symbol: -R 3	Mo *K*α radiation λ = 0.71073 Å
*a* = 12.6562 (18) Å	Cell parameters from 25 reflections
*b* = 12.6562 (18) Å	θ = 10–13º
*c* = 19.696 (4) Å	µ = 0.15 mm^−^^1^
α = 90º	*T* = 293 (2) K
β = 90º	Block, colourless
γ = 120º	0.40 × 0.30 × 0.20 mm
*V* = 2732.2 (8) Å^3^	

### Data collection

**Table:**

Enraf–Nonius CAD-4 diffractometer	*R*_int_ = 0.050
Radiation source: fine-focus sealed tube	θ_max_ = 25.2º
Monochromator: graphite	θ_min_ = 2.1º
*T* = 293(2) K	*h* = −15→7
ω/2θ scans	*k* = 0→15
Absorption correction: ψ scan(North *et al.*, 1968)	*l* = 0→23
*T*_min_ = 0.958, *T*_max_ = 0.971	3 standard reflections
3464 measured reflections	every 200 reflections
1095 independent reflections	intensity decay: none
790 reflections with *I* > 2σ(*I*)	

### Refinement

**Table:**

Refinement on *F*^2^	Secondary atom site location: difference Fourier map
Least-squares matrix: full	Hydrogen site location: inferred from neighbouring sites
*R*[*F*^2^ > 2σ(*F*^2^)] = 0.063	H-atom parameters constrained
*wR*(*F*^2^) = 0.171	*w* = 1/[σ^2^(*F*_o_^2^) + (0.05*P*)^2^ + 4*P*] where *P* = (*F*_o_^2^ + 2*F*_c_^2^)/3
*S* = 1.03	(Δ/σ)_max_ < 0.001
1095 reflections	Δρ_max_ = 0.26 e Å^−^^3^
67 parameters	Δρ_min_ = −0.34 e Å^−^^3^
Primary atom site location: structure-invariant direct methods	Extinction correction: none

### Special details

**Table:**

Geometry. All e.s.d.'s (except the e.s.d. in the dihedral angle between two l.s. planes) are estimated using the full covariance matrix. The cell e.s.d.'s are taken into account individually in the estimation of e.s.d.'s in distances, angles and torsion angles; correlations between e.s.d.'s in cell parameters are only used when they are defined by crystal symmetry. An approximate (isotropic) treatment of cell e.s.d.'s is used for estimating e.s.d.'s involving l.s. planes.
Refinement. Refinement of *F*^2^ against ALL reflections. The weighted *R*-factor *wR* and goodness of fit *S* are based on *F*^2^, conventional *R*-factors *R* are based on *F*, with *F* set to zero for negative *F*^2^. The threshold expression of *F*^2^ > σ(*F*^2^) is used only for calculating *R*-factors(gt) *etc*. and is not relevant to the choice of reflections for refinement. *R*-factors based on *F*^2^ are statistically about twice as large as those based on *F*, and *R*- factors based on ALL data will be even larger.

### Fractional atomic coordinates and isotropic or equivalent isotropic displacement parameters (Å^2^)

**Table:**

	*x*	*y*	*z*	*U*_iso_*/*U*_eq_	
P	0.6667	0.3333	0.01046 (7)	0.0705 (5)	
C1	0.8153 (4)	0.8316 (3)	−0.1198 (2)	0.0992 (12)	
H1A	0.7776	0.8686	−0.0944	0.149*	
H1B	0.7882	0.8209	−0.1661	0.149*	
H1C	0.9024	0.8832	−0.1182	0.149*	
C2	0.7805 (3)	0.7091 (3)	−0.08924 (18)	0.0710 (8)	
C3	0.8232 (3)	0.6365 (3)	−0.11520 (14)	0.0647 (8)	
H3A	0.8752	0.6636	−0.1525	0.078*	
C4	0.7903 (3)	0.5238 (3)	−0.08689 (15)	0.0644 (7)	
H4A	0.8205	0.4768	−0.1058	0.077*	
C5	0.7147 (3)	0.4803 (2)	−0.03192 (14)	0.0609 (7)	
C6	0.6732 (3)	0.5549 (3)	−0.0040 (2)	0.0811 (10)	
H6A	0.6247	0.5301	0.0348	0.097*	
C7	0.7050 (3)	0.6663 (3)	−0.03445 (19)	0.0809 (10)	
H7A	0.6735	0.7130	−0.0167	0.097*	

### Atomic displacement parameters (Å^2^)

**Table:**

	*U*^11^	*U*^22^	*U*^33^	*U*^12^	*U*^13^	*U*^23^
P	0.0791 (6)	0.0791 (6)	0.0533 (8)	0.0396 (3)	0.000	0.000
C1	0.096 (3)	0.076 (2)	0.128 (4)	0.044 (2)	0.000 (2)	0.008 (2)
C2	0.0533 (16)	0.0599 (17)	0.097 (2)	0.0259 (14)	−0.0093 (15)	−0.0109 (16)
C3	0.0609 (16)	0.0699 (18)	0.0587 (17)	0.0294 (14)	0.0003 (13)	−0.0060 (13)
C4	0.0648 (17)	0.0653 (17)	0.0666 (18)	0.0353 (14)	−0.0009 (14)	−0.0129 (14)
C5	0.0607 (16)	0.0677 (17)	0.0530 (16)	0.0312 (13)	−0.0028 (12)	−0.0109 (13)
C6	0.069 (2)	0.083 (2)	0.091 (2)	0.0377 (17)	0.0151 (17)	−0.0131 (18)
C7	0.074 (2)	0.076 (2)	0.100 (3)	0.0436 (17)	0.0036 (18)	−0.0164 (18)

### Geometric parameters (Å, °)

**Table:**

P—C5^i^	1.843 (3)	C3—C4	1.388 (4)
P—C5^ii^	1.843 (3)	C3—H3A	0.9300
P—C5	1.843 (3)	C4—C5	1.366 (4)
C1—C2	1.508 (4)	C4—H4A	0.9300
C1—H1A	0.9600	C5—C6	1.401 (4)
C1—H1B	0.9600	C6—C7	1.394 (5)
C1—H1C	0.9600	C6—H6A	0.9300
C2—C7	1.361 (5)	C7—H7A	0.9300
C2—C3	1.377 (4)		
			
C5^i^—P—C5^ii^	101.08 (11)	C4—C3—H3A	119.3
C5^i^—P—C5	101.08 (11)	C5—C4—C3	121.5 (3)
C5^ii^—P—C5	101.08 (11)	C5—C4—H4A	119.3
C2—C1—H1A	109.5	C3—C4—H4A	119.3
C2—C1—H1B	109.5	C4—C5—C6	117.6 (3)
H1A—C1—H1B	109.5	C4—C5—P	125.2 (2)
C2—C1—H1C	109.5	C6—C5—P	117.1 (2)
H1A—C1—H1C	109.5	C7—C6—C5	119.8 (3)
H1B—C1—H1C	109.5	C7—C6—H6A	120.1
C7—C2—C3	117.4 (3)	C5—C6—H6A	120.1
C7—C2—C1	120.8 (3)	C2—C7—C6	122.3 (3)
C3—C2—C1	121.8 (3)	C2—C7—H7A	118.8
C2—C3—C4	121.4 (3)	C6—C7—H7A	118.8
C2—C3—H3A	119.3		
			
C7—C2—C3—C4	0.3 (5)	C5^i^—P—C5—C6	−169.0 (2)
C1—C2—C3—C4	−179.8 (3)	C5^ii^—P—C5—C6	87.2 (3)
C2—C3—C4—C5	−0.3 (5)	C4—C5—C6—C7	3.0 (5)
C3—C4—C5—C6	−1.4 (5)	P—C5—C6—C7	−179.0 (3)
C3—C4—C5—P	−179.2 (2)	C3—C2—C7—C6	1.5 (5)
C5^i^—P—C5—C4	8.8 (3)	C1—C2—C7—C6	−178.4 (3)
C5^ii^—P—C5—C4	−95.0 (2)	C5—C6—C7—C2	−3.2 (5)

Symmetry codes: (i) −*x*+*y*+1, −*x*+1, *z*; (ii) −*y*+1, *x*−*y*, *z*.
